# Calcium-Dependent Increases in Protein Kinase-A Activity in Mouse Retinal Ganglion Cells Are Mediated by Multiple Adenylate Cyclases

**DOI:** 10.1371/journal.pone.0007877

**Published:** 2009-11-17

**Authors:** Timothy A. Dunn, Daniel R. Storm, Marla B. Feller

**Affiliations:** 1 Neurobiology Section, Division of Biological Sciences, University of California San Diego, La Jolla, California, United States of America; 2 Department of Pharmacology, University of Washington, Seattle, Washington, United States of America; 3 Department of Molecular and Cell Biology and the Helen Wills Neuroscience Institute, University of California, Berkeley, California, United States of America; Harvard University, United States of America

## Abstract

Neurons undergo long term, activity dependent changes that are mediated by activation of second messenger cascades. In particular, calcium-dependent activation of the cyclic-AMP/Protein kinase A signaling cascade has been implicated in several developmental processes including cell survival, axonal outgrowth, and axonal refinement. The biochemical link between calcium influx and the activation of the cAMP/PKA pathway is primarily mediated through adenylate cyclases. Here, dual imaging of intracellular calcium concentration and PKA activity was used to assay the role of different classes of calcium-dependent adenylate cyclases (ACs) in the activation of the cAMP/PKA pathway in retinal ganglion cells (RGCs). Surprisingly, depolarization-induced calcium-dependent PKA transients persist in *barrelless* mice lacking AC1, the predominant calcium-dependent adenylate cyclase in RGCs, as well as in double knockout mice lacking both AC1 and AC8. Furthermore, in a subset of RGCs, depolarization-induced PKA transients persist during the inhibition of all transmembrane adenylate cyclases. These results are consistent with the existence of a soluble adenylate cyclase that plays a role in calcium-dependent activation of the cAMP/PKA cascade in neurons.

## Introduction

During development, many regions of the nervous system exhibit spontaneous biochemical and electrical activity. These early forms of activity, which can occur either on a cell-by-cell basis or correlated across cells by early synaptic connections, play a critical role in various developmental processes in the formation of neural circuits [for review, see1,2]. For example, prior to the onset of vision, retinas exhibit highly patterned, spontaneous activity, termed retinal waves [for review, see 3]. At the level of individual retinal ganglion cells (RGCs), retinal waves drive periodic bursts of depolarization, lasting roughly 2–4 seconds, occurring about once per minute [4], [5].

There is growing evidence that periodic depolarizations in RGCs drive events critical for circuit development via activation of the cAMP/PKA cascade. First, periodic activation of RGCs enhances axonal outgrowth in response to neurotrophins [6], an effect that is blocked by adenylate cyclase inhibitors [7]. Second, periodic increases in cAMP concentration in RGC growth cones are critical for their repulsive response to guidance molecules [8]–[10]. Third, retinal activity is critical for mediating RGC survival, a process that is dependent on cAMP [6], [7].

Recently, we determined that depolarization of RGCs reliably activates a cAMP/PKA second messenger cascade in a calcium-dependent manner [11]. However, the biochemical basis of depolarization-induced increases in PKA activity in RGCs remains unknown. Cyclic AMP levels in neurons are primarily controlled by adenylate cyclases (ACs), which convert ATP to cAMP, and phosphodiesterases (PDEs), which cleave cAMP. Several ACs have been reported to be present in the rodent ganglion cell layer, including calcium activated AC1, AC3, and AC8 [12]. Recent reports suggest that soluble adenylate cyclase (sAC) is also present in some neurons [13]-[16] although its presence in retinal neurons is controversial [16], [17]. Calcium-activated PDE1 is reported to be in the ganglion cell layer [18] but no calcium inhibited PDEs have been described.

Here we used combined calcium and PKA activity imaging [19]–[25], pharmacology and genetically modified mice to determine the roles of specific ACs and PDEs in calcium-dependent PKA activation in RGCs.

## Results

### Combined Calcium and PKA Activity Imaging Reveals Calcium Transients Precede Transient Increases in PKA Activity

**Figure 1:**
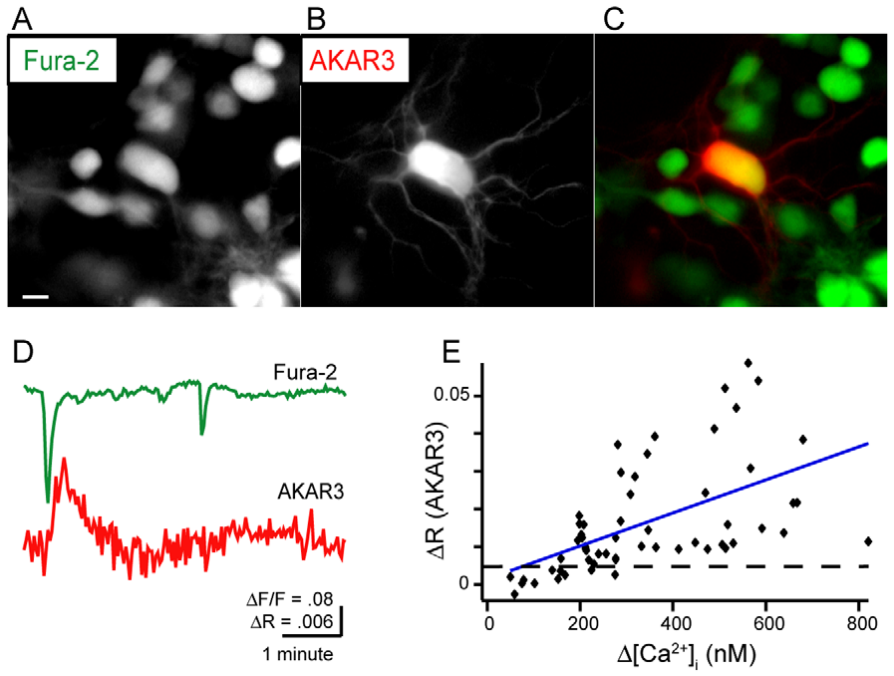
Simultaneous FRET and calcium imaging reveal relationship between depolarization-induced calcium transients and PKA activity. A. Fluorescence image of ganglion cell layer loaded with calcium indicator, fura-2AM. Scale bar  = 10 µm. B. Fluorescence image of RGC expressing the PKA activity indicator AKAR3. C. Pseudocolor representation of two images in A and B. D. Time course of spontaneous changesevents in the fractional change in fura-2 fluorescence recorded simultaneously withspontaneous AKAR3 FRET ratio. FirstThe first spontaneous events revealed that spontaneousa calcium transient preceded a PKA transient, while a smaller calcium transient led to no change in PKA activity. E. Comparison of AKAR3 FRET ratio changes (ΔR) to amplitude of intracellular calcium concentration increase (Δ**[Ca^2+^]_I_**) evoked by short application (duration  = 2 seconds) of high-potassium ACSF. Dashed line: Threshold of detectable FRET ratio change. Blue line: fit of data (r = .551). See Figure S1 for comparison of ΔR vs. fractional change in fluorescence, ΔF/F.

Previously we observed spontaneous and depolarization-induced PKA transients in developing retinal ganglion cells [11]. Here, we used a dual imaging technique to record both spontaneous and evoked changes in calcium levels and PKA activity in RGC somas, where it is likely that multiple pathways for calcium dependent cAMP production will coexist[21]. Plasmids containing the genetically encoded A-kinase activity reporter [19]–[25], AKAR3, under the CMV promoter, were transfected into retinal explants and cultured for 12 hours. To image intracellular calcium concentrations, [Ca^2+^]_I,_ in the same cell, we used the calcium indicator fura-2, which has an excitation wavelength (380 nm) spectrally separate from both CFP (430 nm) and YFP (505 nm). Cells were loaded with membrane permeant fura-2-AM immediately prior to imaging of explants transfected with AKAR3 (Figure 1A–C).

Spontaneous PKA transients were always preceded by large calcium transients associated with retinal waves, as determined by correlated increases in [Ca^2+^]_I,_ in neighboring RGCs (5 transients in 4 cells). However, smaller calcium transients were not followed by PKA transients (see example in Figure 1D). These observations are consistent with previous observations that sustained depolarizations lasting more than 3 seconds were required to reliably induce PKA transients in immature RGCs [11]. Extending the duration of depolarization is more effective than increasing strength of depolarization because immature RGCs are limited in their ability to fire high frequency bursts of action potentials [26]–[28].

The observation that a sustained depolarization was necessary to evoke a detectable PKA transient indicates that a threshold amount of calcium is necessary. To determine this threshold, we compared the amplitude of AKAR3 FRET ratio changes with the increase in [Ca^2+^]_I_ (Figure 1E, Figure S1) evoked by short applications of the high potassium solution (105 mM, 2 sec). We found that ΔF/F>25%, corresponding to an increase an increase of [Ca^2+^]_I_ of 164±7.5 nM, was necessary for inducing a reliable change in the AKAR 3 FRET ratio.

### Mice Lacking Adenylate Cyclase 1 (*Barrelless*) and Adenylate Cyclase 1/8 Double Knockouts Retain Calcium-Dependent PKA Transients

The biochemical link between depolarization and activation of the cAMP/PKA cascade in neurons is thought to be mediated by calcium-dependent adenylate cyclases [29]. Three calcium-dependent adenylate cyclases are expressed in the retina; AC1, AC8 and AC3. AC3 was reported to be stimulated by calcium *in vitro* [30], though recent results suggest that *in vivo*, it acts to decrease production of cAMP in response to calcium influx [31], [32], and therefore is unlikely to mediate the increase in PKA activity following depolarization.

**Figure 2:**
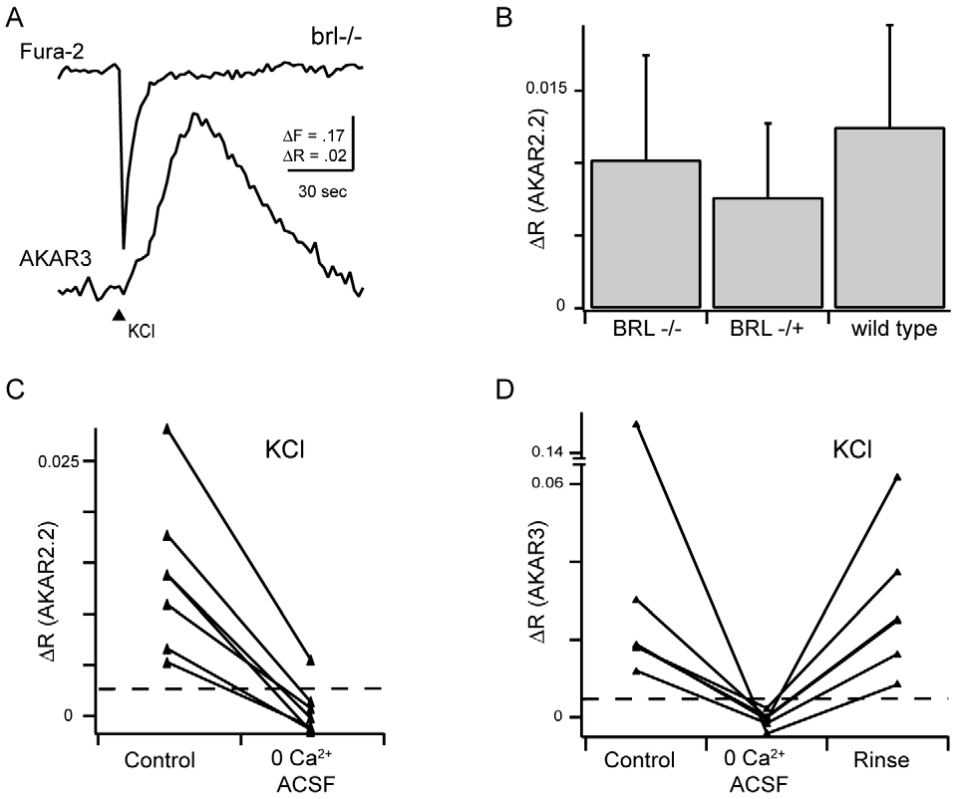
Mice lacking Adenylate cyclase 1 activity exhibited calcium-dependent increases in PKA activity. A. Simultaneous calcium and AKAR3 FRET imaging from a RGC in response to brief depolarization by KCl in *brl* mice, which lack the calcium-dependent adenylate cyclase AC1. B. Summary of AKAR2.2 FRET ratio change amplitudes in response to brief depolarization from *brl* mice, heterozygote littermate controls, and WT mice. Error bars are standard deviation. C. Summary of AKAR2.2 FRET ratio changes in response to depolarization in the presence and absence of extracellular calcium. D. Summary of AKAR3 FRET ratio changes in *brl* mice in response to depolarization in the presence or absence of extracellular calcium. Results do not depend on the PKA activity indicator used.

To address the role of the most prominent AC in the retina, AC1[12], [33], in calcium-dependent PKA activity increases, we utilized a mutant mouse lacking functional AC1, known as *brl*. RGCs from these mice expressing either AKAR2.2 or AKAR3 were depolarized using brief application of high concentration KCl (Figures 2A & B). Note due to its improved sensitivity, FRET ratio changes reported by AKAR3 are substantially larger than those reported in AKAR2.2.

RGCs from *brl* mice were found to retain depolarization-induced PKA transients in all cells assayed, and furthermore, these transients were dependent on the influx of calcium. The amplitude of PKA activity transients generated by depolarization was not significantly different from control or heterozygote littermate controls given the high standard deviations within RGCs of a particular genotype (Figure 2B, *brl*+/- ΔR = 0.0076±0.0051, n = 13 neurons; *brl*-/-: ΔR = 0.010±0.0072, n = 16 neurons, wild-type: ΔR = 0.012±0.0071, n = 12 neurons). Hence, depolarization activates PKA in the absence of AC1.

**Figure 3:**
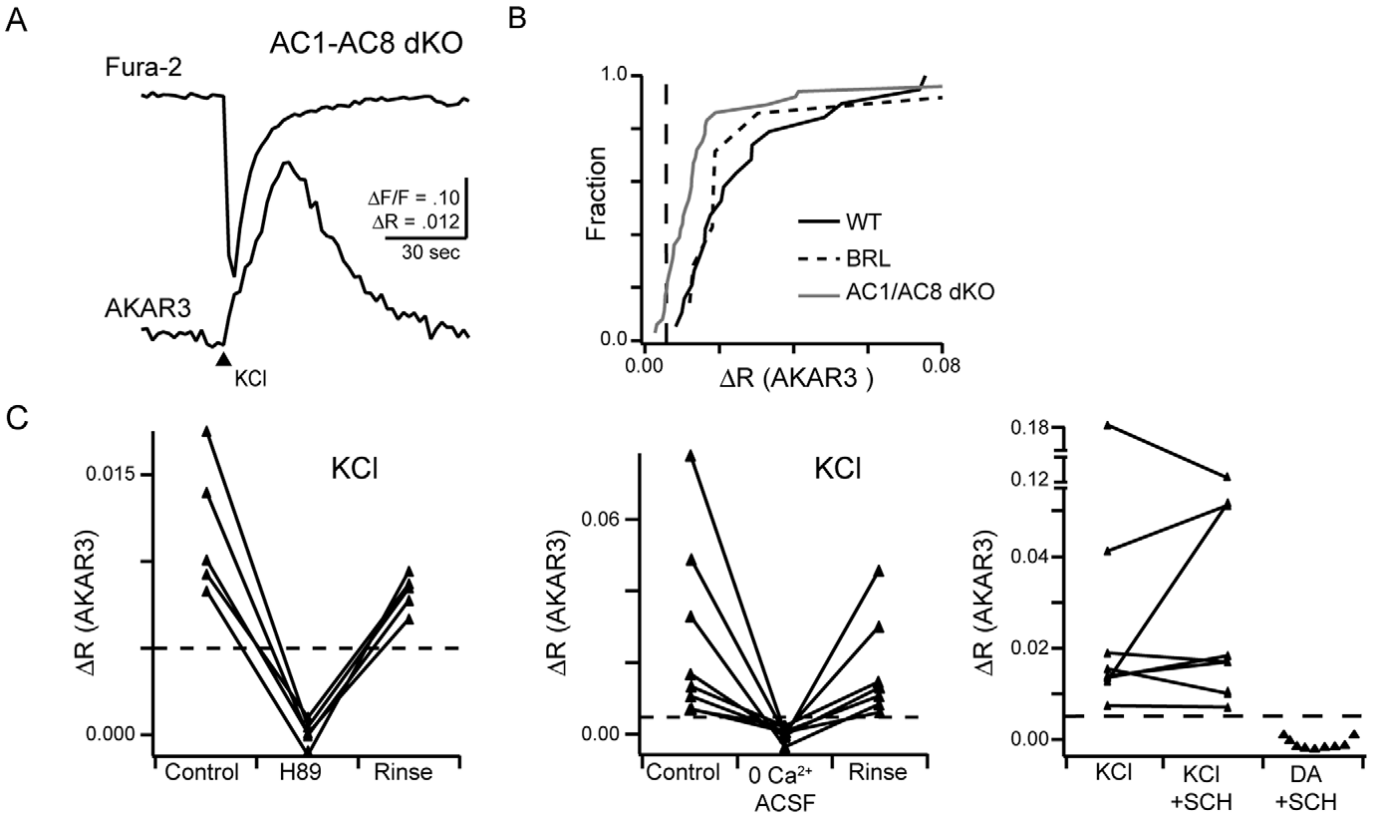
Double knockout mice lacking both Adenylate cyclase 1 and Adenylate cyclase 8 activity exhibit calcium dependent increases in PKA activity. A. Time course of fura-2 fluorescence and FRET ratio change in response to KCl puff in AC1/AC8 dKOs. B. Summary of AKAR3 FRET ratio changes in wild type, AC1 lacking, and AC1/AC8 dKOs. C. Summary of depolarization induced PKA transients in presence or absence of PKA inhibitor H89 (left), external calcium (middle) and the D1-like dopamine receptor antagonist, SCH 23390 (right). As a control, we showed SCH blocked AKAR3 response to short application of dopamine (DA).

A second calcium-dependent adenylate cyclase expressed in RGCs is AC8, though at this stage of developments it is expressed at much lower levels than AC1 [12], [33], [34]. To test for a role for AC8 in mediating calcium-dependent PKA transients, or the potential compensation AC8 for AC1 in the brl mice, we repeated these experiments in double knockout (dKO) mice, lacking both AC1 and AC8 [35], [36]. Surprisingly, the majority of AKAR3-expressing AC1/AC8 dKO RGCs retained depolarization-induced PKA transients (Figure 3A, 28 of 35 cells, n = 29 mice), though they were significantly smaller in amplitude than those observed in brl and WT mice (Figure 3B, median ± standard deviation: wild-type ΔR = 0.021±0.021, n = 19 neurons; AC1/AC8 ΔR = 0.011±0.034, n = 35 neurons, p = 0.0012; *brl* ΔR = 0.018±0.043, n = 7 neurons, p = 0.9999; two tailed Mann-Whitney test). If we eliminate the RGCs that had a response below the detection limit in AC1/AC8 KO mice (Figure 1), the median response was closer to that reported in WT though still statistically significantly lower (ΔR = 0.013+− 0.037, P = 0.015). In contrast, depolarization led to detectable changes in FRET ratios in all wild-type or brl RGCs tested.

We conducted several control experiments on the PKA transients that persisted in AC1/AC8 dKO mice. First, they were blocked by the PKA antagonist H89 (50 µM, 0.5±8.9 percent of control, n = 5 cells from 5 mice, Figure 3C). Second, they were absent in 0-Ca^2+^ ACSF (2.4±12 percent of control, n = 6 cells from 5 mice, Figure 3C). Third, they persisted in the presence of the D1-like dopamine receptor antagonist, SCH 23390 (138±111 percent of control, n = 8 cells from 7 mice, Figure 3C), indicating they were not due to indirect activation of the dopamine receptor via potassium-induced release of dopamine.

Hence, we conclude that the combination of AC1 and AC8 contribute to but are not the exclusive cause of calcium-dependent activation of PKA in the somas of retinal ganglion cells.

We found there was a large variability in the absolutely magnitude of the FRET ratio changes in response to depolarizations. This variability did not correlated with the age at which we transfected retinas or the baseline expression level of the indicators. Rather, we hypothesize that it is due to varied expression of calcium-stimulated ACs and relative levels kinase and phosphatase activities on PKA target substrates.

### Calcium-Dependent PKA Transients Are Retained during Blockade of Transmembrane Adenylate Cyclases

The above findings indicate that the primary identified transmembrane calcium-dependent adenylate cyclases do not fully mediate the depolarization-induced PKA activity in RGCs. Though most ACs in the nervous system are transmembrane, recent reports have indicated that neurons may also have soluble ACs [13]–[15]. Soluble ACs are activated by bicarbonate and calcium, not G proteins. Soluble ACs have been studied in the context of sperm maturation and function [37], [38], but have recently been reported to have a developmental role in the outgrowth of axonsmediate responses to chemical cues present during neural development [13], [14].

**Figure 4:**
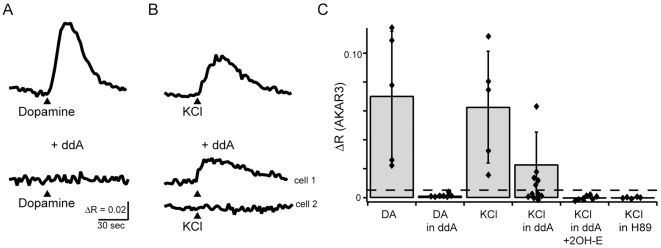
Depolarization-induces increases in PKA activity in a subset of RGCs dependent upon soluble adenylate cyclases. A. Timecourse of AKAR3 FRET ratio in response to stimulation by 2 sec application of dopamine (arrowhead, 50 µM) in absence (top) and presence (bottom) of 2′,5′-dideoxyadenosine (ddA, 50 µM), blocker of transmembrane adenylate cyclases. B. Timecourse of AKAR FRET ratio in response to stimulation by 2 sec application of KCl (105 mM) in absence (top) and presence (bottom) of ddA . Upper example: KCl-induced FRET ratio change in the presence of ddA (5/11 RGCs); Lower example: no FRET ratio change in response to KCl depolarization (6/11 RGCs). C. Summary of FRET ratio responses to short applications of dopamine (DA) or KCl in RGCs expressing AKAR3. In the presence of the combination of ddA (50 µM) and the soluable adenylate cyclase inhibitor 2-OH-E (20 µM) or the specific PKA inhibitor H89 (50 µM), no detectable FRET ratio changes occurred. Error bars are standard deviation.

To differentiate between the activity of calcium-dependent transmembrane ACs and soluble ACs, we utilized 2′,5′-dideoxyadenosine (ddA), a drug that specifically blocks all transmembrane AC activity[13], [39]. First, to determine the effectiveness of ddA in blocking the function of transmembrane ACs in RGCs, we assayed its effects on dopamine-induced elevation of cAMP. Rodent RGCs have D1-like dopamine receptors that elevate cAMP upon activation via transmembrane ACs [40], [41]. Brief application of dopamine (50 µM, 2 sec) led to increases in AKAR3 FRET ratio in all tested RGCs (Figure 4A ΔR = 0.070±0.045, n = 5/5 cells). Dopamine responses were reliably blocked after incubation in ddA (Figure 4A, n = 7 cells).

Second, we assayed the effects of ddA on depolarization-induced PKA transients in RGCs. Depolarization-induced FRET ratio changes were recorded in a subset of RGCs the presence of ddA, though they were reduced in amplitude (Figure 4B, ΔR = 0.023±0.023, n = 5 of 11 cells). Hence, as in the AC1/AC8 dKO mice, in a substantial fraction of cells, blockade of transmembrane ACs decreased but did not prevent activation of cAMP/PKA cascade (Figure 4C).

To test whether the remaining depolarization induced PKA activation was mediated by a soluble AC activity, we repeated the experiment in the presence of ddA and 2-hydroxyestradiol (2-OHE), which is specific for soluble ACs at low concentrations [14], [42]. In the presence of both drugs, depolarization failed to induce an increase the FRET ratio n = 8 cells, Figure 4C). This finding offers the first evidence for the presence of a soluble AC activity in developing RGCs.

### Phosphodiesterases Modulate but Are Not Responsible for the Generation of PKA Transients

Thus far, we have studied the role of adenylate cyclases in depolarization-induced increases in PKA activity in RGCs. However, cAMP levels are also influenced by phosphodiesterases. Indeed, retinal neurons express a calcium-dependent phosphodiesterase that could potentially mediate AC-independent changes in PKA activity [18].

**Figure 5:**
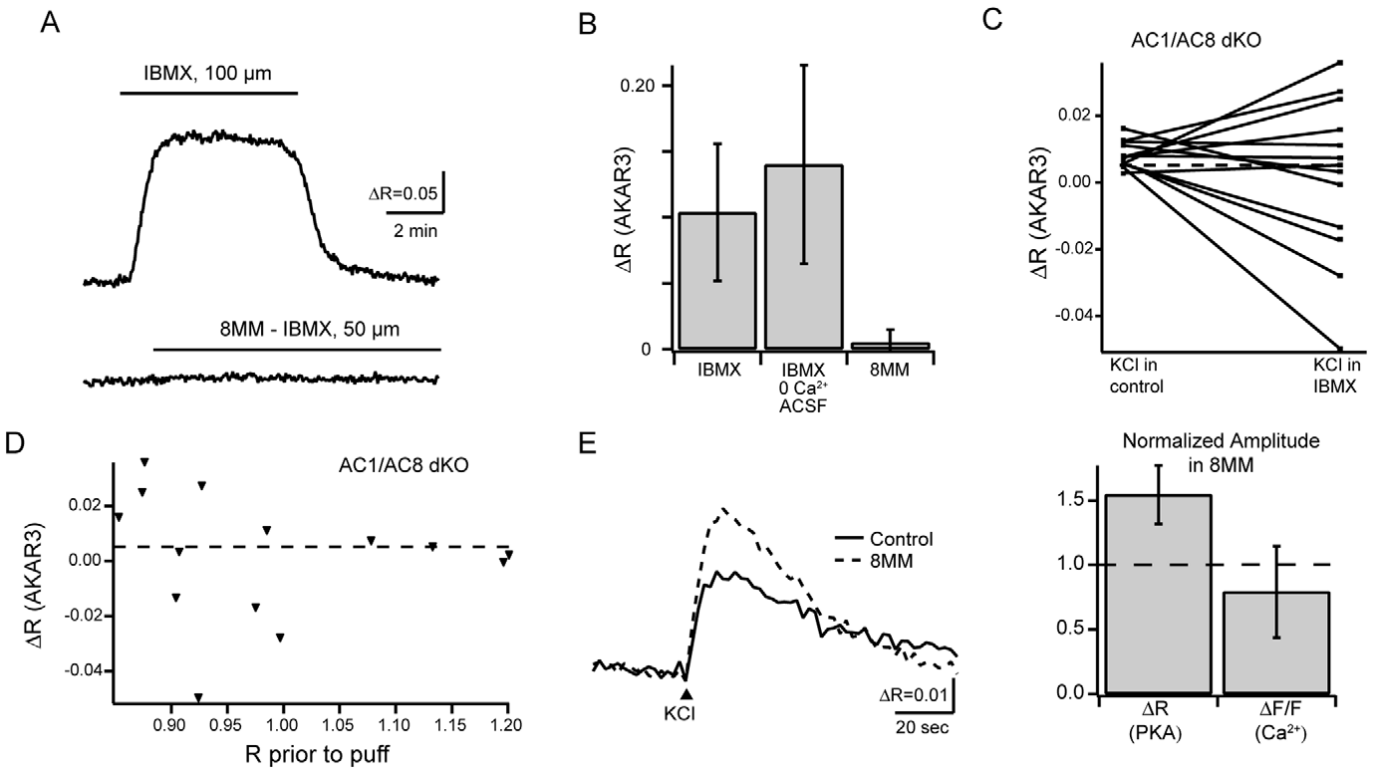
PDEs contribute to, but are not necessary for, the generation of depolarization induced PKA transients. A. AKAR3 FRET ratio traces during bath application of (upper) the general phosphodiesterase inhibitor IBMX (black bar) and (lower) the PDE1 inhibitor, 8MM-IBMX. B. Summary of FRET ratio changes in response to IBMX or 8MM-IBMX. C. Summary of depolarization-induced PKA transients in AC1/AC8 dKOs in the presence or absence of IBMX. Note, in some cases, depolarization led to a decrease in DR.**Δ**R. D. FRET ratio change due to depolarization in the presence of IBMX as a function of ratio prior to depolarization in AC1/AC8 dKOs. E. Left: FRET ratio traces in response to KCl-induced depolarization in the presence or absence of 8MM-IBMX. Right: Summary of FRET ratio changes and fluorescence changes in response to depolarization, while in the presence of 8MM-IBMX. Amplitudes are normalized to changes observed in control conditions for each RGC.

First, to test for a role of phosphodiesterases in generating PKA transients in RGCs, we bath applied the non-specific PDE inhibitor IBMX (100 µM) to retinal explants and monitored PKA activity using AKAR3. IBMX significantly increased the baseline AKAR3 FRET ratio, indicating that PDEs are constitutively active in WT RGCs (Figure 5A ΔR = 0.10±0.052, n = 9 cells in 9 mice) as well as AC1/AC8 dKO RGCs (ΔR = 0.13±0.085, n = 9 cells in 9 mice). This baseline increase in PKA activity in the presence of IBMX persisted in the absence of external calcium (Figure 5B, ΔR = 0.14±0.076, n = 8 cells in 7 mice), indicating that ongoing activity of PDEs sets resting levels of PKA activity in a calcium-independent manner. IBMX however, did not prevent depolarization-induced AKAR3 FRET ratio changes in most WT (ΔR = 0.034±0.021, n = 9 cells in 9 mice) and AC1/AC8 dKO RGCs (ΔR = 0.015±0.011, n = 10 cells in 9 mice). Hence, PDEs are unnecessary for the generation of depolarization induced PKA transients in most RGCs.

In a subset of AC1/AC8 dKO cells (n = 3/10), depolarization in the presence of IBMX did not induce a change in FRET ratio (Figure 5C). Interestingly, in all of these cells, the resting FRET ratio in the presence of IBMX was higher than 1.1, suggesting that the indicator was saturated, i.e. all the indicator was phosphorylated prior to the depolarization (Figure 5D). In addition, in another subset of cells in IBMX (n = 4/14) depolarization induced decreases in the FRET ratio. This decrease may be due to a calcium dependent increase in phosphatase activity, such as calcineurin, that could dephosphorylate AKAR3 directly, lowering the FRET ratio.

To test directly for a role of the calcium-dependent phosphodiesterase PDE1, which is present in the ganglion cell layer [18], in depolarization induced PKA activity, we repeated these experiments in the presence of 8MM-IBMX, a specific inhibitor of calcium-activated PDE1. In contrast to IBMX, 8MM-IBMX did not lead to an increase in the baseline AKAR3 FRET ratio (Figure 5A,B). However, AKAR3 FRET ratio changes were increased in magnitude compared to control depolarization (Figure 5E, ΔR_8MM_/ΔR_con_ = 1.55±0.226, n = 6 neurons, p = 0.002. This increase in PKA activity transients was not due to an increase in calcium transient amplitude (ΔF/F_8MM_/ΔF/F_con_ = 0.79±0.35, n = 6 neurons, p = 0.21). These results imply that although PDEs are not responsible for generating PKA transients in response to depolarization, PDE1 is involved in regulating their amplitude.

## Discussion

Here we have shown that depolarization-induced increases of PKA activity in developing RGC somas are mediated by a combination of transmembrane and soluble calcium-dependent adenylate cyclases. First, dual imaging of a genetically encoded reporter of PKA activity and a membrane permeant form of the calcium dye fura-2 was used to determine that a threshold calcium increase was necessary to induce a measurable PKA transient. Second, depolarization-induced PKA transients were decreased in amplitude but, surprisingly, persisted in knockout mice lacking the calcium dependent adenylate cyclase AC1 and in double knockout mice lacking both AC1 and AC8. Consistent with this finding, depolarization-induced PKA transients persisted in a subset of RGCs in blockers of all transmembrane ACs. The remaining PKA transients were blocked in soluble AC antagonist, providing the first evidence that a soluble AC is present in developing RGCs. Last, blockade of PDEs increased the basal levels of PKA activity and a calcium dependent PDE modulated the amplitude of depolarization-induced PKA transients. Hence, a variety of calcium-dependent ACs and PDEs are important for translating depolarization into transient activation of the PKA biochemical cascade in RGCs.

We imaged PKA activity in the somas of RGCs, where it is likely to influence long-term changes in neurons mediated by transcription. However, there is evidence that in different compartments of neurons, different adenylate cyclase subtypes may be responsible for localized cAMP production and PKA activation [43]. This compartmentalization may be true for RGCs. Our results indicate AC1 Is not responsible for activity-dependent transients in the somas of RGCs, while it is postulated to play a role in activity-dependent elevation of cAMP in growth cones of RGCs, where it mediates repulsive interactions [8].

### Combined Imaging of Calcium and cAMP Is a Powerful Technique for Probing the Intracellular Signaling Pathways of Neurons

Dual imaging of FRET-based cAMP reporters and the spectrally separate calcium indicator fura-2 has recently been used to monitor the intracellular activity of cultured, excitable cells [20], [21], [23]. In this study, we utilize this technique to determine the relationship between calcium transients and PKA activity in RGCs in retinal explants. Dual imaging offers distinct advantages over previous techniques used to correlate PKA with neuronal activity. In the CNS, whole cell recording is often used to monitor the activity of single neurons. However, this technique requires the disruption of the membrane of the neuron, which in turn dialyzes the contents of the cell with the solution in the electrode, which interrupt second-messenger signaling. Indeed, whole cell recordings from RGCs prevented FRET ratio changes even in response to strong stimuli like forskolin [11]. Second, it allows for simultaneous detection of intracellular calcium concentration and cAMP/PKA activity – two pathways whose dynamic interactions strongly regulate cell function [44].

Dual imaging of AKAR3 and fura-2, allowed us to determine several aspects of the relationship between calcium transients and PKA activity on a cell-by-cell basis. First, calcium transients reliably preceded all PKA activity transients. This is consistent with the hypothesis that calcium/calmodulin stimulated ACs are being activated to increase cAMP levels. Second, a threshold increase in [Ca^2+^]_I_ of approximately 165 nM was necessary for inducing a detectable PKA transient. The observation that increases in [Ca^2+^]_I_ around 200 nM can lead to large increases in the AKAR ratio (Figure 1E and Figure S1) indicate that this threshold represents a feature of the biochemical pathway rather than the sensitivity of AKAR3. Third, though there was a small correlation between the amplitude of FRET ratio changes and calcium transients, there was a large variability in FRET ratio changes for a given calcium transient amplitude (Figure 1E). This variability was found throughout the experiments described in the paper, making it difficult to find statistical differences between groups of cells. We hypothesize that this variability reflects biological differences in cells, such as varied expression of calcium-stimulated ACs. One caveat is that AKAR3 reports the relative level of kinase and phosphatase activities on PKA target substrates, so if phosphatase activity is slowly varying over time, it may be responsible for some of the variance of FRET ratios we see in response to calcium influx.

### Multiple Calcium-Stimulated Adenylate Cyclases Are Responsible for Calcium Dependent PKA Activity in RGCs

Our findings indicate that no single AC underlies all calcium-dependent PKA transients in developing RGCs. Using pharmacological blockade of a certain class of ACs, we found that roughly half of RGCs rely entirely on transmembrane ACs to produce calcium dependent PKA transients (Figure 4). This finding is consistent with our observations that mice lacking both AC1 and AC8, the dominant calcium-dependent ACs in RGCs, continue to have calcium-dependent increases in PKA activity (Figures 2 and 3). We observed that *brl* mice, which lack AC1, do not have significantly altered PKA transients, and that double knockouts of AC1/AC8 have smaller transients. We cannot tell whether this difference implies that AC8 is more likely to play an important role in calcium-dependent PKA activation or whether there was compensation by overexpression of AC8 in the *brl* mouse. These findings indicate that RGCs exhibit a ddA-insensitive AC activity, likely soluble AC, as this activity was abolished when an inhibitor of sACs was included. Intriguingly, intact hippocampal neurons, but not isolated membranes, lacking AC1 and AC8 also exhibit a calcium-dependent adenylate cyclase activity, suggesting that a soluble AC activity is present in other CNS regions [15].

### The Role of Phosphodiesterases in Transient Activation of PKA in RGCs

Our results provide several insights into the activity of PDEs in early postnatal RGCs (Figure 5). First, blockade of PDEs reveals a constitutive AC activity indicating that PDEs are also constitutively active in maintaining a basal level of cAMP in the cell. Second, we rule out the possibility that PDEs are involved in the generation of transient PKA activity in both wild-type and AC1/AC8 dKO. Finally, we report that blockade of calcium stimulated PDE1 leads to increased amplitude of FRET ratio changes in response to depolarization. This suggests that PDE1 plays a role in decreasing cAMP levels after calcium influx in RGCs.

In summary, we have provided evidence that in developing retinal ganglion cells, depolarization modulates the cAMP/PKA pathways via a variety of calcium-dependent biochemical cascades. Identifying these various points of modulation is critical for elucidating the mechanisms by which neural activity influences the development of neural circuits.

## Materials and Methods

### Mutant Mouse Strains

Mice were handled and housed in accordance with the University of California, Berkeley Institutional Animal Care and Use Committee and IACUC guidelines. Mice lacking functional AC1 through a spontaneous mutation, known as *barrelless* mice [45]–[47], were a generous gift from M. Crair. (Yale University). They came from the eighth backcross generation of the incipient C57BL/6J-Adcy1 (*barrelless, brl*) congenic inbred strain. AC1 wild type (Wt) and heterozygous (*brl* ^+/−^) mice were used as controls. AC1/AC8 double knockout mice bred into C57BL/6 background for more than eight generations were generated as described previously [35], [36]. Genotypes were determined by genomic PCR using primer sequences described previously [36], [48].

### Retinal Explants

The methods for generating and transfecting explants are described in full in Dunn and Wang, et al., 2006. Briefly, P0-P3 mouse retinal explants from wild-type, *barrelless*, or AC1/AC8 double knockout (dKO) mice were transfected using electroporation with AKAR2.2 or AKAR3, genetically encoded indicators of PKA activity (plasmids for both indicators from J. Zhang) [19], [22], [24], [25], [49], [50].

Retinal explants were isolated and mounted on filter paper with the RGCs facing up. Then, 0.2 mg/mL plasmid was electroporated (30 V, 4 mm, 2 pulses at 1 second interval, BTX ECM 830 electroporator). Retinal explants were then cultured in serum free culture medium (Neurobasal-A medium supplemented with B27 (Gibco), 0.6% glucose, 2 mM glutamine, 10 mM HEPES, 1 mM Na-Pyruvate, 50 mg/mL penicillin G, 50 units/mL streptomycin, 2.5 mg/mL Insulin, 6 µM forskolin, 10 ng/mL CNTF & 50 ng/mL BDNF) for 12–48 hours. Explants were removed to forskolin-free media overnight prior to imaging. Immediately prior to imaging, the explants were placed in serum free culture media containing 10 µM fura-2-AM, 1% DMSO, and 0.02% pluronic acid for 30–60 min.

### Dual Imaging of FRET-Based Indicators and Calcium

FRET imaging was performed on an upright Zeiss Axioskop 2, using a 60x LUMPLFLW water immersion objective (Olympus), and perfused continuously with warmed (30–34°C) artificial cerebrospinal fluid (ACSF: 119 mM NaCl, 2.5 mM KCl, 1.3 mM MgCl_2_, 1.0 mM K_2_HPO_4_, 2.5 mM CaCl_2_, 26.2 mM NaHCO_3_, 11 mM D-glucose) bubbled with 95% O_2_/5% CO_2_. The filter configuration was similar to previous studies of simultaneous calcium and FRET imaging [21], [51]. For FRET imaging, CFP was excited by a narrow bandwidth 436/10 nm filter to minimize direct excitation of fura-2. Two emission wavelengths were collected simultaneously by using a Dual-View image splitter (Optical Insights) with appropriate yellow (535 nm/30) and cyan emission filters (480 nm/40). Calcium imaging was performed using fura-2-AM, with excitation at 380 nm and a 455DCLP dichroic mirror (Chroma). The peak emission for fura-2 occurs at 510 nm, but the emission band is broad enough that both our yellow and cyan filters passed fura-2 fluorescence. Therefore, fluorescence intensity collected by both emission filters was combined to comprise the total fura-2 emission. Images were captured with a CCD camera (CoolSnap HQ, Photometrics) and subsequently analyzed (MetaMorph v6.3, Universal Imaging). Images of CFP and YFP emission were acquired at a rate of 0.5 Hz, with exposure times ranging from 100 ms to 1 sec, determined by the baseline expression of indicators. Images of fura-2 emission were acquired consecutively following each FRET image.

To measure the observed intensity of the reporters in individual cells, regions of interest were drawn around the entire soma of a RGC expressing AKAR, and the average intensity of CFP, YFP and fura-2, less the background, was recorded for each time point. Cells were selected on the following criteria: 1) RGCs were identified by the presence of fluorescence in an axon at the surface of the retina; 2) the baseline expression of indicators was roughly 3 times above background for 1 second exposures; 3) there was evidence of a baseline FRET interaction between CFP and YFP in the unstimulated state and 4) the cell has a detectable FRET ratio change in response to either KCl depolarization, forskolin or dopamine stimulation. The following observed intensities were recorded: 1) I_CFP_, when excited by 436 nm light and collected by the CFP emission filter, 2) I_YFP_, when excited by 436 nm light and collected by the YFP emission filter, 3) I_fura,c_, when excited by 380 nm light and collected by the CFP emission filter and, 4) I_fura,y_ when excited by 380 nm light and collected by the YFP emission filter. Each of these observed intensity values contain bleedthrough from the other indicator. Bleedthrough constants were established through separate experiments in RGCs expressing a single fluorophore. These values were used to determine the corrected fluorescence values F_CFP_, F_YFP_, F_fura,c_, and F_fura,y_ as explained below.

To measure FRET ratios, bleedthrough of CFP and fura-2 were corrected. CFP bleedthrough into the YFP channel was determined to be 45%. Fura-2 bleedthrough upon CFP excitation was 8.2% in the CFP channel and 9.8% in the YFP channel. Intensity of the FRET fluorophores was corrected as follows:
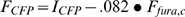


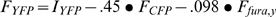



FRET ratios were then calculated as R = (F_YFP_/F_CFP_). Both AKAR2.2 and AKAR3 report increases in PKA activity as increases in FRET ratio, though the FRET ratio changes reported by AKAR3 are substantially larger than those reported in AKAR2.2. Changes in FRET ratio, ΔR = (F_YFP_/F_CFP_)_manipulation_-(F_YFP_/F_CFP_)_control_, due to drug application or depolarization were computed by subtracting the average value of the FRET ratio over 5 frames prior to the stimulus from the average value of the FRET ratio over 5 frames at the maximum response within 60 seconds following the stimulus.

To analyze fura-2 fluorescence intensity, F_fura_, fura-2 emission from both channels was summed, and bleedthrough from CFP was corrected. Excitation at 380 nm for calcium imaging led to excitation of CFP at 9% efficiency of the preferred 436 nm excitation with negligible excitation of the YFP fluorophore. Therefore, all fura-2 fluorescence traces were corrected for these bleedthrough factors using the following algorithm:
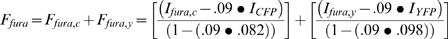



Amplitudes of calcium events were calculated as ΔF/F by using a five-frame average fura-2 intensity as the baseline, and the single frame with the minimum fura-2 intensity.

To correlate the changes in PKA activity as ascertained by changes in the FRET ratio with changes in intracellular calcium concentration, [Ca^2+^]_I_, we used the following method. We first estimated the resting [Ca^2+^]_I_ using ratiometric fura-2 measurements and the following equation: 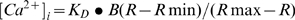
where R_min_, R_max_ and B, were determined *in vitro* [52]. We recorded the following values: R_min_ = 0.119, R_max_ = 7.27, and B = 15.2. We assumed a K_D_ for fura-2 in cells of 224 nM [52]. Retinal ganglion cells electroporated without plasmid, cultured for 24 hours and incubated with fura-2-AM had a resting calcium concentration of 226±21 nM (n = 12). The increases in [Ca^2+^]_I_ induced by short applications of potassium (see Figure 1) were calculated using the following equation:
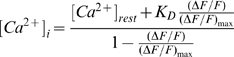
where [Ca^2+^]_rest_ is the resting calcium concentration and ΔF/F_max_ is based on calibrations with excitation at 380 nm [52].

An important concern when imaging a three-fluorophore system is to correctly adjust the raw data for bleedthrough and unintentional excitation of the other fluorophores. However, we found that taking the ratio of YFP/CFP was remarkably effective at eliminating the contribution of fura-2 fluorescence, leading to minimal changes (<10%) in ΔR amplitude after correcting for bleedthrough. Also, because the timing of the images of calcium levels and PKA activity was not simultaneous, we were unable to completely eliminate the bleedthrough of fura-2 during rapid fluorescence changes, particularly during the first frame of a calcium event.

*Pharmacology*:

All pharmacological agents were purchased from Sigma. Puffing solution contained 5 mM KCl, 123 mM NaCl, 3 mM CaCl_2_, 2 mM MgCl_2_, 10 mM glucose and 10 mM HEPES, pH was adjusted with NaOH to 7.3. For dopamine application, freshly prepared dopamine (50 µM) was added to puffing solution. High potassium puffing solution was similar, with 100 mM KCl substituted for an equivalent amount of NaCl, leading to a final concentration of 105 mM KCl inside the puffing electrode. One to three second applications of dopamine or high potassium solution were delivered through a glass pipette (approximately 2 µm tip size, located within 20 microns from transfected neuron) using a PV830 Pneumatic PicoPump at 6–8 psi, 50 msec pulse duration at 4 Hz. The duration of dopamine or potassium application was altered by increasing the number of pulses. For experiments with zero external calcium, ACSF was modified by replacing 2.5 mM CaCl_2_ with 2 mM EGTA and 1.7 mM MgCl_2._

We utilized 2′,5′-dideoxyadenosine (ddA), a drug that specifically blocks transmembrane AC activity via the P site [13], [39]. Since application of ddA required an extensive incubation period, the effects of ddA on dopamine and KCl responses were tested on separate population of cells than the control experiment.

## Supporting Information

Figure S1 Simultaneous FRET and calcium imaging reveal relationship between depolarization-induced PKA activity transients and amplitude of calcium transients. A) Comparison of AKAR3 FRET ratio changes to amplitude of calcium transients evoked by potassium induced depolarization of RGCs. A ΔF/F of −20% represents a calcium influx of 164 nM while a −60% change represents a calcium influx of 807 nM (see Figure 1). Blue line: Linear fit of data (r = −.589).
(0.07 MB DOC)
