# Supplementary material for: Calcium-Dependent Increases in Protein Kinase-A Activity in Mouse Retinal Ganglion Cells Are Mediated by Multiple Adenylate Cyclases
Source: PLoS One. 2009 Nov 17;4(11):e7877. doi: 10.1371/journal.pone.0007877 (PMC2774513; doi:10.1371/journal.pone.0007877)
Supplement: Figure S1 — Simultaneous FRET and calcium imaging reveal relationship between depolarization-induced PKA activity transients and amplitude of calcium transients. A) Comparison of AKAR3 FRET ratio changes to amplitude of calcium transients evoked by potassium induced depolarization of RGCs. A ΔF/F of −20% represents a calcium influx of 164 nM while a −60% change represents a calcium influx of 807 nM (see Figure 1). Blue line: Linear fit of data (r = −.589). (0.07 MB DOC) [file pone.0007877.s001.doc]

**
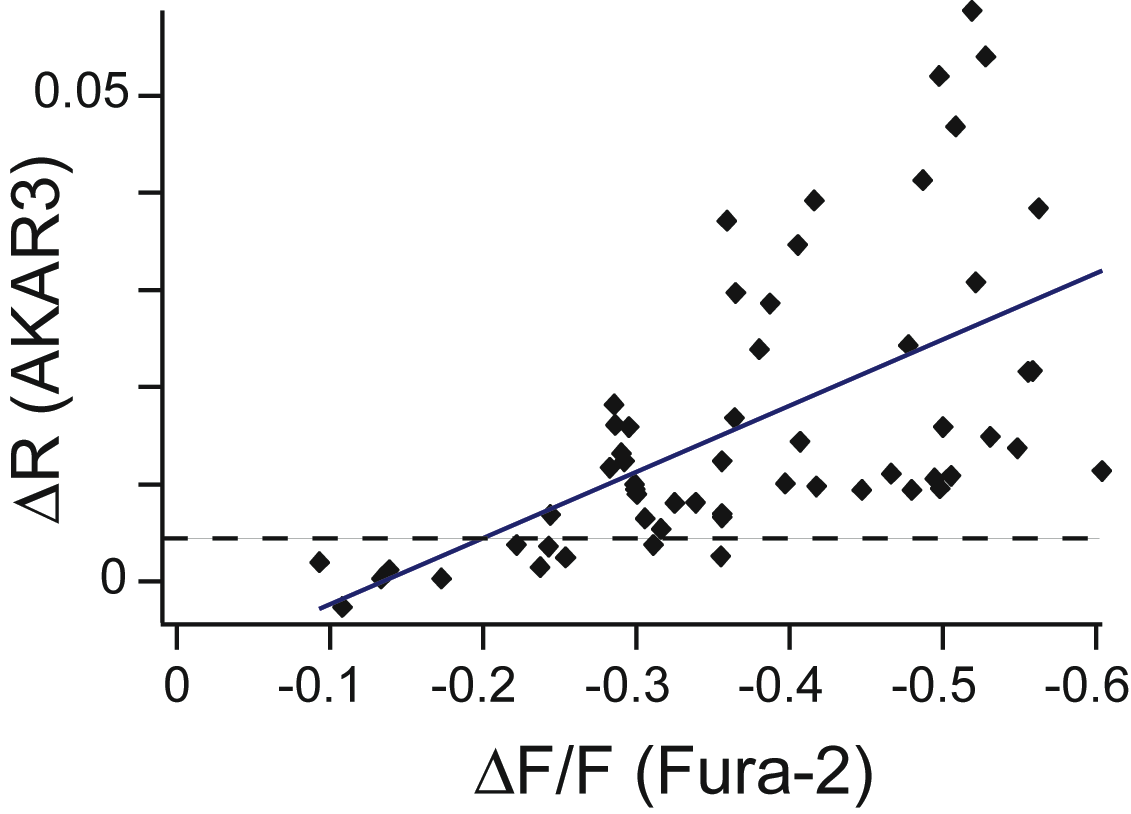
**

**Figure S1. Simultaneous FRET and calcium imaging reveal relationship between depolarization-induced PKA activity transients and amplitude of calcium transients.** A) Comparison of AKAR3 FRET ratio changes to amplitude of calcium transients evoked by potassium-induced depolarization of RGCs. A ΔF/F of –20% represents a calcium influx of 164 nM while a –60% change represents a calcium influx of 807 nM (see Figure 1). Line: Linear fit of data (r=-.589).
